# Ovarian Reserve and ART Outcomes in Blepharophimosis-Ptosis-Epicanthus Inversus Syndrome Patients With *FOXL2* Mutations

**DOI:** 10.3389/fendo.2022.829153

**Published:** 2022-04-28

**Authors:** Tingting Meng, Wenzhe Zhang, Rongrong Zhang, Jie Li, Yuan Gao, Yingying Qin, Xue Jiao

**Affiliations:** ^1^ Center for Reproductive Medicine, Shandong University, Jinan, China; ^2^ Key Laboratory of Reproductive Endocrinology of Ministry of Education, Shandong University, Jinan, China; ^3^ Shandong Key Laboratory of Reproductive Medicine, Shandong University, Jinan, China; ^4^ Shandong Provincial Clinical Research Center for Reproductive Health, Jinan, China; ^5^ National Research Center for Assisted Reproductive Technology and Reproductive Genetics, Shandong University, Jinan, China; ^6^ Suzhou Research Institute, Shandong University, Jinan, China

**Keywords:** blepharophimosis-ptosis-epicanthus inversus syndrome (BPES), *FOXL2*, infertility, ovarian reserve, assisted reproductive technology (ART)

## Abstract

**Objective:**

To characterize the status of ovarian reserve and ART outcomes in BPES women and provide informative reference for clinical diagnosis and treatment.

**Methods:**

Twenty-one women with BPES were screened for mutations in the *FOXL2* gene and underwent assisted reproductive technology (ART) treatment. Indicators for ovarian reserve and ART outcomes were compared between patients with and without *FOXL2* mutations. Additionally, ART outcomes were compared among patients with different subtypes of *FOXL2* mutations.

**Results:**

A total of 13 distinct heterozygous variants in the *FOXL2* gene were identified in 80.95% of BPES women, including 4 novel mutations with plausible pathogenicity (c.173_175dup, c.481C>T, c.576del and c.675_714del). Compared to non-mutation group, patients with *FOXL2* mutations had elevated levels of FSH (P=0.007), decreased AMH levels (P=0.012) and less AFC (P=0.015). They also had worse ART outcomes with large amount of Gn dosage (P=0.008), fewer oocytes (P=0.001), Day3 good quality embryos (P=0.001) and good quality blastocysts (P=0.037), and a higher cancellation rate (P=0.272). High heterogeneity of ART outcomes existed in BPES patients with different *FOXL2* mutation types.

**Conclusions:**

BPES patients with *FOXL2* mutations had diminished ovarian reserve and adverse ART outcomes. The genotype-reproductive phenotype correlations were highly heterogeneous and cannot be generalized. Genetic counseling for fertility planning and preimplantation or prenatal genetic diagnosis to reduce offspring inheritance are recommended.

## Introduction

Blepharophimosis-ptosis-epicanthus inversus syndrome (BPES, OMIM #110100) is a rare autosomal dominant genetic disorder with an estimated prevalence of 1/50,000 (1). It is characterized by narrow horizontal palpebral fissures, ptosis, epicanthus inversus and telecanthus. In addition to isolated ophthalmic features (type II BPES), BPES patients may also present with female infertility or ovarian dysfunction associated with premature ovarian insufficiency (POI), classified as type I BPES (2).

Several pathogenic or candidate genes of BPES have been found, including *FOXL2*, *UBE3B*, *KAT6* and *ITGB5* (3, 4). Approximately 70% of BPES is contributed by heterozygous variations in *FOXL2* gene (5, 6). This single-exon gene encodes a forkhead transcription factor highly conserved among species and predominantly expressed in developing eyelid mesenchyme and fetal and adult ovaries. To date, more than 270 variants in the *FOXL2* gene have been reported to be associated with BPES. Of these, intragenic mutations accounted for 80% and could be subdivided into frameshift mutations (44%), in-frame changes (33%), nonsense (12%) and missense mutations (11%) (5, 7). However, a genotype-phenotype correlation remains elusive given that ovarian function is largely unavailable. A tendency was observed that truncated mutations before the polyalanine tract were preferentially associated with BPES type I, whereas polyalanine expansion variants frequently led to BPES type II (5). Nonetheless, the phenotype associated with missense substitutions and with mutations leading to a truncated or extended protein with an intact forkhead domain and polyalanine tract is not clearly predictable. Furthermore, high heterogeneity existed in BPES patients, either with interfamilial or intrafamilial genetic or phenotypic variability (8). Therefore, the correlation between the *FOXL2* genotype and BPES reproductive phenotype warrants further investigation in larger cohorts.

*FOXL2* is involved in fetal development as well as maintenance of the mature ovary. In the postnatal ovary, it supports follicular growth (9). *FOXL2* deficiency in mice led to atresia of the oocytes with no maturation of secondary follicles. Mutations in the *FOXL2* gene may affect the proliferation, differentiation and steroidogenesis of granulosa cells by inhibiting the transcription of aromatase, P450scc and Cyclin D2, leading to oocyte atresia and progressive follicular depletion in the absence of functional granulosa cells (10–12). Female patients carrying *FOXL2* mutations represent a challenge for genetic counseling in terms of both fertility planning and offspring inheritance. However, the reproductive characteristics in reproductive-aged BPES women were only reported by case reports, including at most 3 cases (13–15). Few studies have previously summarized the clinical, hormonal, and ovarian profiles in BPES women with *FOXL2* mutations, let alone the commonly accepted fertility recommendation and practical intervention for successful pregnancy and healthy offspring.

In the current study, we characterized the ovarian reserve status and assisted reproductive technology (ART) outcomes and further evaluated the genotype-reproductive phenotype correlations in female BPES patients with *FOXL2* mutations, which will provide informative references for the clinical diagnosis, fertility counseling and treatment of BPES patients.

## Materials and Methods

### Patients

A total of 21 Han Chinese women with a clinical diagnosis of BPES were recruited from the Reproductive Hospital Affiliated to Shandong University between April 2018 and March 2021. The typical features of eyelid deformities included bilateral dysplasia of the eyelids with narrow horizontal palpebral fissures (blepharophimosis), droopy upper lids reducing the vertical palpebral aperture (ptosis), bilateral skin fold arising from the medial lower eyelid ascending to the upper lid (epicanthus inversus), and an increased distance between the medial canthi (telecanthus). The diagnostic criteria of POI included oligo/amenorrhea for at least 4 months, and elevated FSH level >25 IU/L (on two occasions >4 weeks apart). Subjects with bilateral fallopian tube obstruction, a history of ovarian surgery, chemo- or radiotherapy, and male factor infertility were excluded. The standardized evaluation consisted of clinical history, physical examination, reproductive characteristics, and genetic and iatrogenic factors. The study was approved by the Institutional Review Board of Center for Reproductive Medicine, Shandong University ([2018]- NO.34). Written informed consents were obtained from all participants.

### Hormone Measurement and Ultrasonography

Peripheral blood was sampled on day 2-4 of menstrual cycle. Endocrine hormones follicle-stimulating hormone (FSH), luteinizing hormone (LH), estradiol (E2), and testosterone (T) were detected through chemiluminescence immunoassay (Roche Diagnostics), and anti-Müllerian hormone (AMH) was detected by enzyme-linked immunosorbent assay (Kangrun Biotech). The intra- and inter-assay coefficients of variation were <10% and <15%, respectively. Transvaginal ultrasonography was routinely conducted. Antral follicle count (AFC) was defined as the number of bilateral follicles (2-10 mm in diameter) in early follicular phase.

### Karyotype Analysis and Mutation Screening in the *FOXL2* Gene

All patients underwent karyotype analysis and exon sequencing of the *FOXL2* gene. Karyotype analysis was performed on GTG-banded metaphase chromosomes prepared from peripheral lymphocyte cultures using a standard protocol that generated 400-450 band resolutions, as detailed in our previous study (16). Chromosome polymorphisms were recorded but classified as normal. For the mutation screening of the *FOXL2* gene, genomic DNA was extracted from the peripheral blood samples using QIAamp DNA Blood Kits (Qiagen). The entire coding sequence and its splice site junctions of *FOXL2* gene were amplified by the polymerase chain reaction (PCR) with overlapping sets of primers designed by Primer Premier 5.0 software: FOXL2-1F (5’-GCAGTCTGGCTTCCTCAACAA-3′), FOXL2-1R (5’-AGGGGACAAAGAGGAGCGAC-3′), FOXL2-2F (5’-CTGCGAAGACATGTTCGAGAAG-3′), and FOXL2-2R (5’-GGACAAAGAGGAGCGACAGG-3′). The PCR products were first analyzed by agarose gel electrophoresis, purified by polyethylene glycol precipitation and then sequenced on an ABI 3730XL DNA Analyzer (Applied Biosystems, Foster City, CA) with the BigDye Terminator Cycle Sequencing Kit (Applied Biosystems, Carlsbad, CA, USA). The results were analyzed using Sequencer version 4.9 software, and the variants identified were compared from the gnomAD, ExAC and 1000 Genomes database. All novel variants were validated by three independent PCRs and bidirectional sequencing. The nomenclature of the mutations identified was based on Human Genome Variation Society guidelines. Amino acid conservation was analyzed by multiple sequence alignment by ClustalW software, and the plausible pathogenic effect of these mutations was classified according to American College of Medical Genetics and Genomics/Association for Molecular Pathology (ACMG/AMP) guidelines and analyzed by PolyPhen-2 and Mutation Taster.

### Controlled Ovarian Hyperstimulation (COH) and ART Procedure

Patients enrolled in the present study received a standardized ovarian stimulation regimen, including gonadotropin releasing hormone (GnRH) agonist long protocol, GnRH agonist short protocol, and GnRH antagonist protocol. The starting dosage was determined by age, ovarian reserve, body mass index (BMI), and previous response to COH. When at least one dominant follicle reached 18 mm in diameter, human chorionic gonadotropin (hCG) at a dose of 6,000-10,000 IU was used for oocyte maturation triggering. Oocyte retrieval was transvaginally performed 36-38 h after hCG injection. Oocytes were fertilized through either conventional insemination or intracytoplasmic sperm injection (ICSI), depending on semen parameters. *In vitro* maturation (IVM) was performed for immature oocytes, including those at either the germinal vesicle (GV) or metaphase I (MI) stage. The IVM culture medium was prepared and balanced overnight one day in advance, and then 10 ng/ml epidermal growth factor (EGF) (Sigma, USA), 75 U/L FSH (Serono, Switzerland) and 75 U/L LH (Serono, Switzerland) were added 4 h before immature oocytes were available. Then the number of metaphase II (MII) stage oocytes with the extrusion of the first polar body was counted. For patients requiring granulosa cell coculture, the cumulus granulosa cells were isolated from the cumulus-oocyte complexes (COCs) by digestion with 80 IU/mL hyaluronidase, cut into small pieces at 500 μm with the microscope, and then washed in the IVM medium. After washing, the cells were added for immature oocyte culture in an incubator at 37°C in 6% CO_2_.

### Outcome Measures

Fertilization was assessed 17-19 h after insemination and defined by the presence of two pronuclei (2PN) or two polar bodies (2PB). Embryo development and quality were assessed 68-72 h (day 3) after insemination based on the number of blastomeres, blastomere symmetry, percentage of fragmentation, and quality of cytoplasm. Day 3 good quality embryo was defined as a minimum of seven blastomeres on day 3 according to the criteria established by the Istanbul Consensus Workshop on Embryo Assessment (17). Day 5/6 embryos were graded using the Gardner scoring system with respect to the inner cell mass and trophectoderm, in which blastocysts equal to or greater than grade 4 BC were defined as good quality blastocysts (18, 19).

### Statistical Analysis

SPSS 23.0 (SPSS Inc., Chicago, IL) was used for data analysis. The Shapiro-wilk test was used for normality of distribution. Continuous variables in normality distribution were expressed as mean ± standard deviation and compared by Student’s *t*-test or one-way analysis of variance. Continuous variables that were not normally distributed were presented as median (quartile interval) and compared by nonparametric test. Categorical variables were presented as percentage (n/N) and compared by chi-square or Fisher’s exact test. A two-tailed value of P<0.05 was considered statistically significant.

## Results

### Baseline Characteristics

A total of 21 participants were recruited with an average age of 30 years old (29.91 ± 4.27 yrs). They showed cardinal features of eyelid deformities from birth with variable severity, including shortened palpebral fissures, drooping eyelids and a tiny skin fold arising from the lower eyelid and an increased distance between the medial canthi. Ten patients (47.62%) had a family history of BPES. All showed infertility (76.2% in primary infertility) with an average duration of 5 years (5.17 ± 3.58 yrs), except for one fertile fetus with 3 fetuses positive for maternal *FOXL2* mutations (patient #12). Menstruation irregularity was found in 57.14% of cases, with one presenting with secondary amenorrhea. Seventeen patients had at least one or more abnormal hormone profile(s), with either elevated FSH (>10 IU/L) and/or decreased AMH (<1.2 ng/mL). However, none of them reached the diagnostic criteria for POI. All patients had normal 46, XX karyotype.

### Molecular Analysis of *FOXL2* Mutations

**Figure 1:**
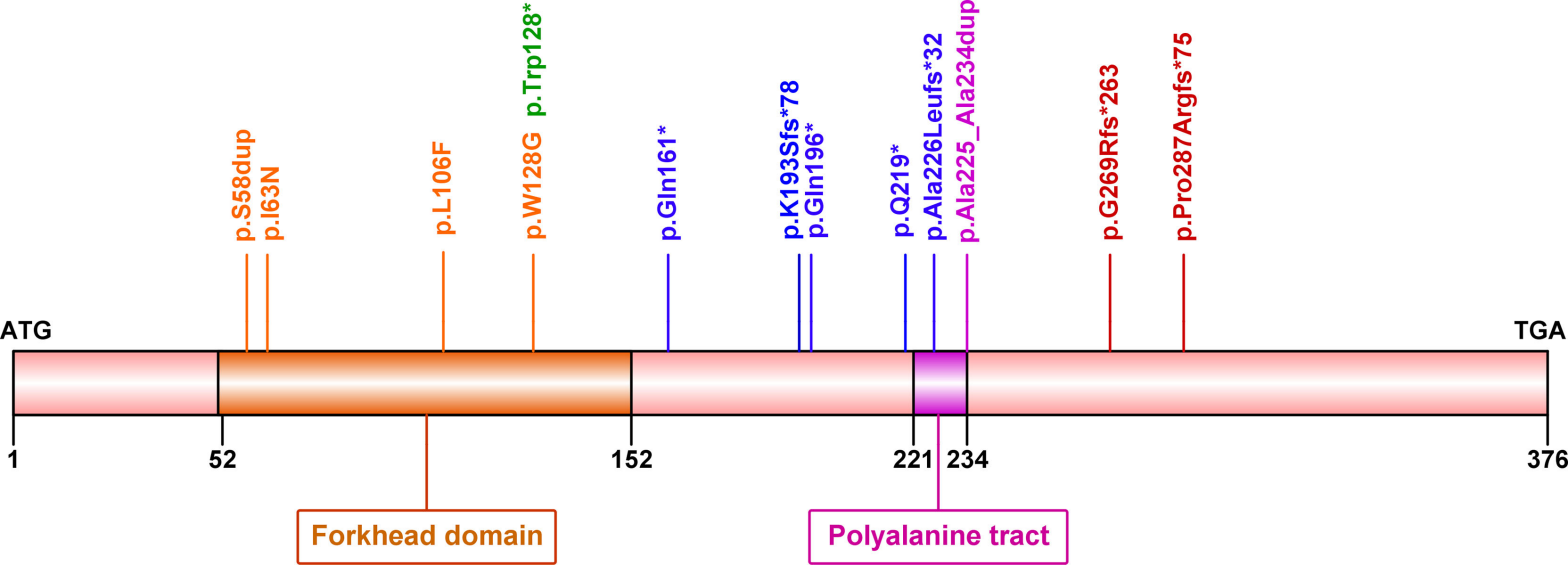
Diagram showing forkhead box L2 (*FOXL2*) protein structure and mutations identified in the current study. Human *FOXL2* encodes a 376 amino acid protein, containing a DNA-binding forkhead domain (amino acid 52–152) and a polyalanine tract (amino acid 221–234).

Among 21 women with BPES, 17 (80.95%) cases carried mutations in the *FOXL2* gene. A total of 13 distinct heterozygous variants were identified, including 2 in-frame duplications, 3 missense variants, 4 nonsense and 4 frameshift variants (**Table 1** and **Figure 1**). These variant sites were highly conserved among species. Of note, four novel mutations were also identified in 4 patients: c.173_175dup (p.Ser58dup), truncated mutations c.481C>T (p.Gln161*), c.576del (p.Lys193Serfs*78) and c.675_714del (p.Ala226Leufs*32) (5, 20–29). None was found in the gnomAD, ExAC, or 1000 Genomes databases or previously reported in the literature. All were predicted to be potentially pathogenic by PolyPhen-2 or Mutation Taster and classified as pathogenic (P) or likely pathogenic (LP) according to ACMG/AMP guidelines.

**Table 1:** The *FOXL2* mutations identified in the current study.

DNA sequence variation	Amino acid change	Predicted protein change	Inheritance	Allele Frequency	Polyphen-2	Mutation Taster	ACMG	Reference
c.173_175dup	p. Ser58dup	Insertion mutation	Sporadic	0	–	Disease causing	LP	Current study
c.188T>A	p. Ile63Asn	Missense mutation	Familial	0	Probably damaging	Disease causing	LP	(20)
c.316C>T	p. Leu106Phe	Missense mutation	Familial	0	Possibly damaging	Disease causing	LP	(5, 21)
c.382T>G	p. Trp128Gly	Missense mutation	Familial	0	Probably damaging	Disease causing	LP	(22)
c.383G>A	p. Trp128*	Nonsense mutation	Familial	0	–	Disease causing	P	(22, 23)
c.481C>T	p. Gln161*	Nonsense mutation	Sporadic	0	–	Disease causing	LP	Current study
c.576del	p. Lys193Serfs*78	Frameshift mutation	Sporadic	0	–	Disease causing	LP	Current study
c.586C>T	p. Gln196*	Nonsense mutation	Sporadic	0	–	Disease causing	P	(24)
c.655C>T	p. Gln219*	Nonsense mutation	Familial	0	–	Disease causing	P	(25, 26)
c.672_701dup	p. Ala225_Ala234dup	Extended polyalanine tract	Familial	4.28E-05	–	Disease causing	P	(27, 28)
c.675_714del	p. Ala226Leufs*32	Frameshift mutation	Sporadic	0	–	Disease causing	P	Current study
c.804dupC	p. Gly269Argfs*263	Frameshift mutation	Sporadic	0	–	Disease causing	P	(27, 29)
c.843_859dup	p. Pro287Argfs*75	Frameshift mutation	Sporadic	0	–	Disease causing	P	(27)

ACMG, American College of Medical Genetics; LP, likely pathogenic; P, pathogenic.

### Ovarian Reserve in BPES Patients With and Without *FOXL2* Mutations

The status of ovarian reserve was compared between BPES patients with and without *FOXL2* gene mutations (**Table 2**). There was no significant difference in age at diagnosis, BMI or duration of infertility between the two groups (P>0.05). The levels of FSH (17.08 ± 12.74 IU/L vs. 6.66 ± 1.86 IU/L, P=0.007) and LH (11.00 ± 5.76 IU/L vs. 3.23 ± 1.56 IU/L, P=0.017) were significantly higher in patients with *FOXL2* mutations than in those without mutations. Consistently, patients with *FOXL2* mutations also had significantly decreased AMH concentration (0.460 [0.170, 0.858] ng/mL vs. 2.295 [1.120, 4.518] ng/mL, P=0.012) and AFC (8.18 ± 5.13 vs. 15.25 ± 1.71, P=0.015). In addition, we found that 76.47% of patients in the mutation group had irregular menstruation or amenorrhea, while regular menstrual cycles were reported in all four patients without *FOXL2* mutations (P=0.012).

**Table 2:** The ovarian reserve in BPES patients with and without *FOXL2* mutations.

Characteristics	Mutation group	Nonmutation group	P
**N**	17	4	–
**Age (y)**	30.06 ± 4.66	29.25 ± 2.22	0.742
**BMI (kg/m^2^)**	24.81 ± 3.34	24.18 ± 0.23	0.716
**Duration of infertility (y)**	5.29 ± 3.71	4.63 ± 3.38	0.746
**FSH (IU/L)**	17.08 ± 12.74	6.66 ± 1.86	0.007
**LH (IU/L)**	11.00 ± 5.76	3.23 ± 1.56	0.017
**E2 (pg/mL)**	38.86 ± 20.22	29.33 ± 16.27	0.396
**T (ng/dL)**	28.22 ± 14.46	14.84 ± 11.73	0.149
**AMH (ng/mL)**	0.460 (0.170, 0.858)	2.295 (1.120, 4.518)	0.012
**AFC**	8.18 ± 5.13	15.25 ± 1.71	0.015
**Prevalence of irregular menstruation, % (n/N)**	76.47% (13/17)	0 (0/4)	0.012

Data are expressed as the mean ± standard deviation or median (interquartile range).

BMI, body mass index; FSH, follicle-stimulating hormone; LH, luteinizing hormone; E2, estradiol; T, testosterone; AMH, anti-müllerian hormone; AFC, antral follicle count.

### ART Outcomes in BPES Patients With and Without *FOXL2* Mutations

For these 21 women with BPES, 17 ART cycles in total were performed, among which 7 were cancelled due to poor ovarian response (POR). The cycle characteristics and reproductive outcomes of ART were shown in **Table 3**. The gonadotropin (Gn) starting dosage (229.17 ± 54.18 IU vs. 172.50 ± 22.36 IU, P=0.042), Gn duration (15.08 ± 4.54 days vs. 10.00 ± 1.73 days, P=0.014) and total Gn dosage (4926.25 ± 2073.73 IU vs. 2002.50 ± 648.22 IU, P=0.008) in the mutation group were significantly higher than those in the nonmutation group. Furthermore, patients with *FOXL2* mutations had significantly lower level of E2 on trigger day (1553.63 ± 1067.67 pg/ml vs. 2768.00 ± 1039.91 pg/ml, P=0.048) and decreased number of follicles above 14 mm in diameter on the trigger day (4.83 ± 3.97 vs. 14.60 ± 6.19, P=0.001), as well as a significantly lower number of retrieved oocytes (2.75 ± 1.91 vs. 12.40 ± 6.19, P=0.001) than those without mutations.

**Table 3:** The cycle characteristics and reproductive outcomes of ART in BPES patients with and without *FOXL2* mutations.

Characteristics	Mutation group	Nonmutation group	P
**N**	**12**	**5**	
**Gn starting dosage (IU)**	229.17 ± 54.18	172.50 ± 22.36	0.042
**Gn duration (days)**	15.08 ± 4.54	10.00 ± 1.73	0.014
**Total dosage of Gn (IU)**	4926.25 ± 2073.73	2002.50 ± 648.22	0.008
**E2 on trigger day (pg/mL)**	1553.63 ± 1067.67	2768.00 ± 1039.91	0.048
**No. of follicles >14mm on trigger day**	4.83 ± 3.97	14.60 ± 6.19	0.001
**No. of retrieved oocytes**	2.75 ± 1.91	12.40 ± 6.19	0.001
**No. of metaphase II oocytes**	0.5 (0,3)	6 (3,16)	0.006
**Maturation rate, % (n/N)**	51.52% (17/33)	70.97% (44/62)	0.060
**No. of fertilized oocytes**	1 (0,2)	6 (2,8.5)	0.019
**Fertilization rate, % (n/N)**	42.42% (14/33)	43.55% (27/62)	0.916
**No. of day 3 good-quality embryos**	0 (0,0)	4 (1.5,4.5)	0.001
**Day 3 good-quality embryos rate, % (n/N)**	21.43% (3/14)	59.26% (16/27)	0.021
**No. of good-quality blastocysts**	0 (0,1)	2 (0.5,4)	0.037
**Good-quality blastocysts rate, % (n/N)**	28.57% (4/14)	40.74% (11/27)	0.443
**Cancellation rate, % (n/N)**	36.84% (7/19)	0 (0/5)	0.272

Data are expressed as the mean ± standard deviation or median (interquartile range).

For reproductive outcomes related to oocyte quality, fertilization and embryo quality, we found that the number of MII oocytes (0.5 [0, 3] vs. 6 [3, 16], P=0.006) and fertilized oocytes (1 [0, 2] vs. 6 [2,8.5], P=0.019) in the mutation group were significantly decreased compared with those in nonmutation group, as well as the number of day 3 good quality embryos (0 [0,0] vs. 4 [1.5, 4.5], P=0.001) and good quality blastocysts (0 [0,1] vs. 2 [0.5, 4], P=0.037). Consistently, the maturation rate and day 3 good-quality embryo rate were also decreased in patients with *FOXL2* mutations. Additionally, cancelled cycles due to poor ovarian response were present only in the mutation group, although the difference in cycle cancellation rate did not reach statistical significance (36.84% [7/19] vs. 0 [0/5], P=0.272).

### ART Outcomes in BPES Patients With Different Types of *FOXL2* Mutations

**Figure 2:**
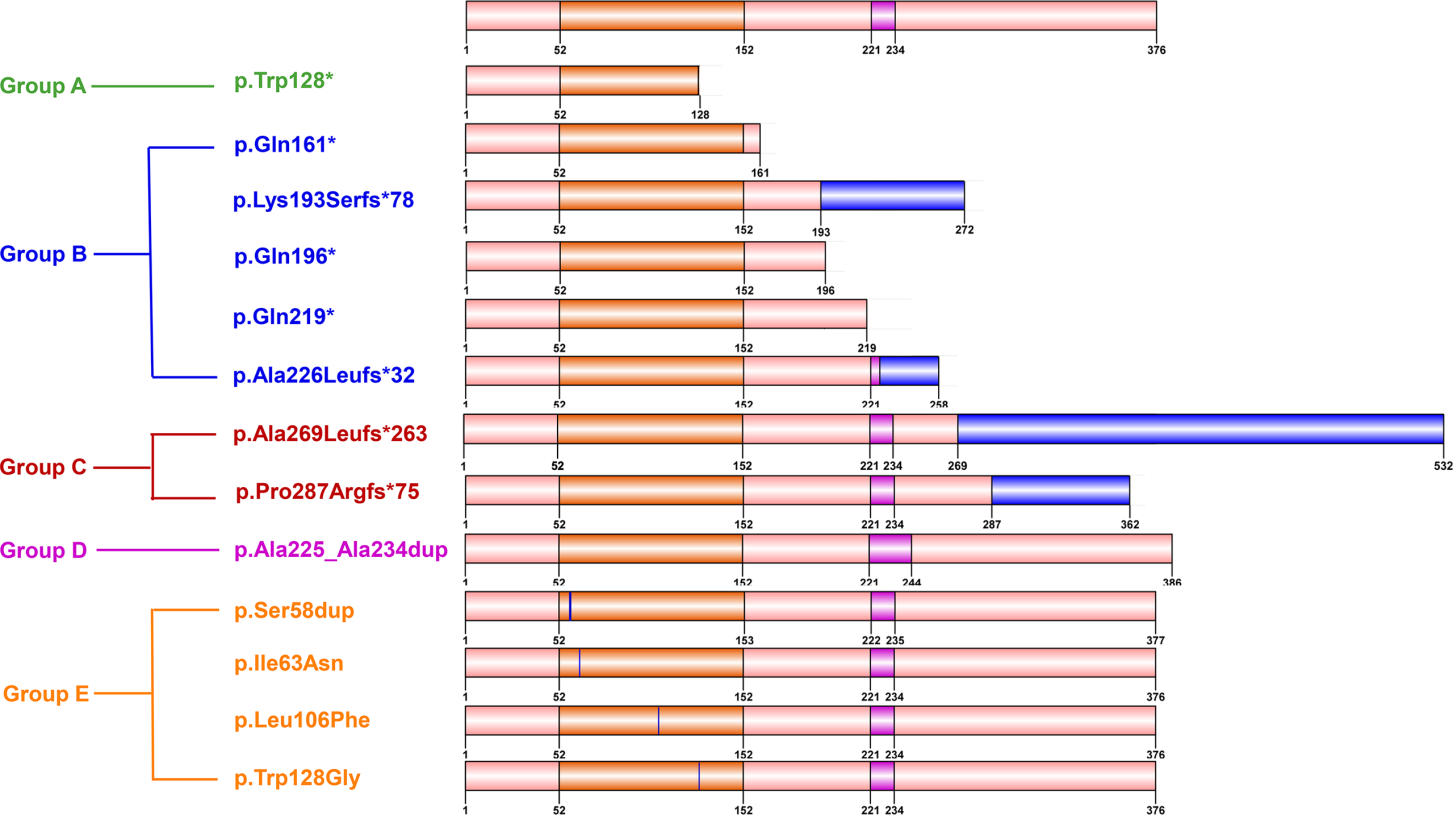
Diagram showing five groups with different types of *FOXL2* mutations. Groups A to C: truncated proteins, with partial forkhead domain **(**Group A**)**, with complete forkhead and without complete polyalanine tract **(**Group B**)**, and with complete forkhead and polyalanine domain **(**Group C**)**. Group D: duplication mutations leading to elongated polyalanine tract, group E: missense and single amino acid insertion mutations in forkhead domain.

To identify a possible genotype-reproductive phenotype correlation, the identified *FOXL2* mutations were further classified into five groups according to their location and the predicted effect on protein. Groups A to C present truncated proteins, with a partial forkhead domain (A), with a complete forkhead and without complete polyalanine domain (B), and with a complete forkhead and polyalanine domain (C). Group D comprised duplication mutations leading to elongated polyalanine tracts, and group E contained missense and single amino acid insertion mutations in the forkhead domain. (**Figure 2** and **Table 4**).

**Table 4:** The ART characteristics and outcomes in BPES patients with different types of *FOXL2* mutations.

Group	Patient ID	Cycle	Gn starting dosage (IU)	Gn duration (days)	Total dosage of Gn (IU)	E2 on trigger day (pg/mL)	No. of follicles >14mm on trigger day	No. of retrieved oocytes	No. of metaphase II oocytes	No. of fertilized oocytes	No. of day 3 good-quality embryos	No. of good-quality blastocysts	No. of good-quality embryos
**A**	**#1**	1	200	18	5250	2301.0	6	3	0	2	0	1	1
	**#2**	1	200	16	4525	3082.0	9	6	0	0	0	0	0
		2	225	17	5315	1473.0	5	4	3	2	0	1	1
		3	300	14	4575	739.0	1	1	1	0	0	0	0
**B**	**#3**	1	Cancelled cycle due to POR										
	**#4**	1	Cancelled cycle due to POR										
	**#5**	1	300	9	2700	856.2	4	0	0	0	0	0	0
		2	225	11	2925	600.0	2	3	3	2	2	0	2
		3	Cancelled cycle due to POR										
	**#6**	0	Preparing for ART treatment currently										
	**#7**	1	Cancelled cycle due to POR										
		2	Cancelled cycle due to POR										
		3	Cancelled cycle due to POR										
**C**	**#8**	0	Egg donation										
	**#9**	0	Egg donation										
**D**	**#10**	1	275	18	6300	533	2	1	0	0	0	0	0
	**#11**	0	Cancelled cycle due to POR										
	**#12**	1	300	10	4350	1011.0	1	3	3	3	0	0	0
		2	150	10	2250	1692.0	4	5	4	2	1	2	2
**E**	**#13**	0	Preparing for ART treatment currently										
	**#14**	1	225	14	3750	3623.0	13	3	0	0	0	0	0
	**#15**	1	200	23	8250	399.4	1	0	0	0	0	0	0
	**#16**	1	150	21	8925	2334.0	10	4	3	3	0	0	0
	**#17**	0	Egg donation										

In group A, the patient #1 and #2 were siblings. They both showed primary infertility (~ 4 yrs) and diminished ovarian reserve (DOR) with elevated FSH (>10 IU/L) and decreased AMH (<1.2 ng/ml). They experienced 4 ART cycles in total. Undergoing one ART cycle, patient #1 had 3 oocytes retrieved but failed pregnancy with one good-quality blastocyst transferred. Among the 3 ART cycles for patient #2, only one high-quality blastocyst was obtained, and a baby girl with a maternal *FOXL2* mutation was born after frozen embryo transfer.

Group B consisted of 5 infertile patients with DOR (FSH >10 IU/L or AMH <1.2 ng/ml). A total of 8 ART cycles were performed, of which 6 were cancelled due to POR. Two high-quality embryos were obtained for patient #5 within 2 cycles, but no pregnancy was achieved after fresh embryo transfer.

Group C included 2 women with primary infertility and the worst ovarian reserve status (patient #8 and #9). Both of them had FSH >15 IU/L, AMH <1.2 ng/ml and AFC <5 simultaneously. They underwent at least 2 COH cycles without dominant follicles available in other hospitals, and hence receiving oocyte donation was recommended.

Three patients were included in group D. Patients #10 and #11 were infertile with DOR (FSH >15 IU/L and AMH <1.2 ng/ml), turned out without available embryo with single oocyte retrieved or ART cycle canceled due to POR. Patient #12 had normal fertility but multiple offspring with *FOXL2* mutations. She had 4 natural pregnancies, including 1 BPES daughter and 3 abortions due to positivity for *FOXL2* mutations in the fetus by prenatal diagnosis. Consequently, she asked for fertility counseling and underwent 2 cycles of preimplantation genetic test (PGT) in our center. In the first cycle, 3 immature oocytes were retrieved and matured *in vitro* by coculturing with granulosa cells but subsequently degenerated after fertilization. In the second cycle, 4 oocytes were retrieved and matured *in vitro* with granulosa cells from normal donors, which resulted in 2 high-quality blastocysts. However, neither was transferred owing to carrying maternal pathogenic mutations indicated by PGT.

There were 5 patients in group E, of which patients #15 and #17 were sisters. All 5 patients had relatively normal ovarian reserve indicated by FSH and AMH, and two of them (patient #13 and #15) experienced natural pregnancies. Of these, 3 patients (patient #14, #15 and #16) underwent 3 ART cycles, except for #17 with donor eggs. For patient #15, no oocytes were retrieved except 3 empty mucus masses within one cycle. In the ART cycle of patients #14 and #16, the oocytes retrieved with poor oocyte-corona-cumulus complexes remained immature or degenerated after IVM, and consequently no transferable embryos were available.

In summary, among 17 BPES patients with *FOXL2* mutations, 4 had transferable embryos available but clinical pregnancy with maternal *FOXL2* mutation was achieved in one case.

## Discussion

BPES is a rare genetic disorder characterized by eyelid manifestation in the presence or absence of infertility or ovary dysfunction, with *FOXL2* as the dominant pathogenic gene. Currently, the genotype-reproductive phenotype correlation remains elusive. In the current study, we identified 13 distinct *FOXL2* heterozygous variants in 6 BPES families and 7 sporadic cases, of which 4 were novel with potentially pathogenic effects. The ovarian reserve in BPES patients with *FOXL2* gene mutations was significantly more impaired than that in those without mutations. Furthermore, BPES patients with *FOXL2* mutations had worse ART outcomes, characterized by a large Gn dosage, fewer oocytes and high-quality embryos, and a higher cycle cancellation rate. High heterogeneity existed in the ART outcomes of BPES patients with distinct mutation types. We did not discover more grievous reproductive impairments related to *FOXL2* mutations with more severe deleterious functional effects. Furthermore, for women with BPES with *FOXL2* mutations, PGT is recommended to reduce the risk of offspring inheritance.

Since the BPES classification was first proposed in 1983, an increasing number of studies on *FOXL2* mutations and functional experiments have been reported (2). The majority focused on ophthalmology or included mainly children and male patients, and reproductive characteristics have seldom been comprehensively evaluated in reproductive-aged women except for some case reports (15, 25, 30, 31). Here, we are the first to characterize fertility, ovarian reserve status and ART outcomes in BPES women with *FOXL2* mutations. We found that the ovarian reserve, quality of oocytes and embryos, and reproductive outcomes were significantly compromised in BPES women positive for *FOXL2* mutations compared to those without mutations, although they all presented with infertility. In the adult ovary, *FOXL2* is predominately expressed in the undifferentiated granulosa cells of the growing follicles and affects granulosa cell proliferation, differentiation and steroidogenesis by inhibiting the activity of steroidogenic acute regulatory (StAR) protein, aromatase, P450scc and Cyclin D2 (9–11, 32). Ablation of *FOXL2* could result in female infertility with follicles blocked between the primordial and primary stages and subsequent follicle atresia. Therefore, the impairment of reproductive phenotypes of our patients could be contributed by the functional deficiency of *FOXL2* in ovarian development and functional maintenance.

Notably, unlike the severe ovarian phenotype in *FOXL2* knockout mice, patients with heterozygous *FOXL2* mutations usually undergo complete follicle development and normal menarche, followed by oligomenorrhea and amenorrhea due to accelerated depletion of the follicle pool and subsequent ovarian dysfunction or even POI. The BPES patients with *FOXL2* mutations in our study were infertile due to ovarian dysfunction with variable severity, but none exhibited POI until now. Inappropriate luteinization of graafian follicles might be the major pathophysiological mechanism involved. Alternatively, the patients are still younger than 40 years old and they might suffer from the more severe phenotype of POI later. In addition, *FOXL2* could also be expressed in the adult pituitary and essential for *FSHB* transcription in the pituitary and for maintaining the FSH levels required (33, 34). Defects in FSH secretion resulting from the *FOXL2* mutations might partially explain the low FSH levels in most BPES patients (35). Given that, the classification of BPES should not merely rely on the presence of abnormal FSH levels but focus on whether there is intact ovarian function or fertility.

The establishment of a genotype-phenotype correlation is of great importance for fertility prediction and timely intervention for reproductive-aged women. To date, it remains a great challenge to distinguish BPES types I and II and ovarian dysfunction with varied severity based on different *FOXL2* mutations. It is widely believed that truncations before the polyalanine tract, inducing loss of the C-terminal region acting as a transcriptional inhibitory domain, are preferentially associated with BPES type I (5, 36–38). Consistently, five truncations p. Trp128*, p. Gln161*, p. Lys193Serfs*78, p. Gln196* and p. Ala226Leufs*32 were first reported to be related to BPES type I in our study. Interestingly, we also found truncated mutations of p. Gly269Argfs and p. Pro287Argfs*75 after the polyalanine tract, which have been strongly associated with BPES type I (6, 29, 39, 40). Polyalanine expansion mutations, acting as hypomorphic alleles and retaining partial transactivation effects, are commonly considered to lead to BPES type II (5, 36, 41). However, the mutation p. Ala225_Ala234dup, which has previously been reported in patients with BPES type II, was detected for the first time in our patient with BPES type I. The ovarian phenotype might relate to the abnormal nuclear aggregation and cytoplasmic mislocalization of mutated *FOXL2* proteins (42, 43). Missense mutations were considered particularly associated with typical palpebral malformations independent of ovarian phenotypes. Intriguingly, the 3 missense mutations previously reported in BPES type II were also found in our type I patients. These mutations have been shown to have subcellular mislocalization and adverse impacts on the transactivation of *CYP19A1*, *CCND2*, and *StAR* by *in-vitro* functional experiments (7, 20–22). Therefore, the genotype-phenotype BPES classification correlation cannot be generalized. In addition, high phenotypic variability existed within *FOXL2* mutations of the same domain and even within the same mutation, although with the presence of ovarian dysfunction. In our study, although with the same c.672_701dup mutation, patient #12 was fertile with normal ovarian reserve, while patients #10 and #11 exhibited infertility (>5 yrs duration) and a DOR phenotype, both of whom had FSH >15 IU/L, AMH <1.2 ng/ml and AFC ≤5 simultaneously. Of note, when we classified different *FOXL2* mutations found in our BPES type I patients from severe to mild predicted functional impairment (from group A to E), we unfortunately did not find more severe reproductive phenotypes associated with more serious functional defects. Taken together, the clinical findings of our patient confirm phenotypic overlap and question a clear-cut prediction of female fertility based on *FOXL2* molecular defects only. The wide clinical variability observed indicates that the reproductive phenotype of *FOXL2* mutations may depend not only on the causal mutation but also on other contributing factors, including unknown modifier genes, epigenetics and/or environmental factors. Of note, although our study is the largest study describing the ART outcomes in BPES women currently, the sample size is small and additional studies in larger cohorts are required to establish clear-cut genotype-phenotype correlations.

The genetic counseling approach should be routinely established in clinics for BPES patients. First, *FOXL2* mutation testing in BPES women of childbearing age should be handled with caution, given that genotype-phenotype correlation cannot be generalized. Nonetheless, based on our data, BPES patients with *FOXL2* gene mutations had reduced fertility and decreased ovarian reserve, and they might have a higher risk for POI than mutation-negative BPES patients. Risk evaluation and surveillance and effective and timely fertility intervention are recommended before POI finally occurs. Furthermore, BPES has a high familial heritability with an inheritance risk of 50%. In our study, patient #12 had multiple offspring or pregnancies with *FOXL2* mutations, including 1 BPES daughter, 3 abortions indicated by prenatal diagnosis, and 2 PGT embryos, which brought her great physical and psychological trauma. Therefore, prenatal genetic counseling for offspring health is also particularly important and highly recommended for BPES patients with *FOXL2* mutations. For adolescent girls, delayed development of secondary sexual characteristics and menarche should be considered. For reproductive-aged women, ovarian function should be monitored, and fertility planning is recommended for future risk of POI. For those with natural pregnancy, prenatal diagnosis through amniotic fluid or fetal umbilical cord blood should be performed, whereas for those with infertility, ART treatment, PGT for normal and high-quality embryo screening, or egg donation are recommended.

Of note, BPES patients with *FOXL2* mutations frequently suffer from failure of IVF pregnancy due to poor oocyte quality with compromised developmental potential, even after IVM. The hypothesis is that the inherent functional defects of granulosa cells due to *FOXL2* mutations might not provide an optimal microenvironment for the growth and maturation of oocytes. Currently, there is no consensus or recommendation to improve reproductive success for BPES patients with *FOXL2* mutations. We assume that remedial supplementation or coculturing of granulosa cells with immature oocytes during IVM treatment would improve oocyte quality by simulating or improving the microenvironment of oocyte maturation, and establishing communication with oocytes through gap junctions (44). For patient #12, two blastocysts were obtained after coculturing granulosa cells from normal donors. These preliminary data to some extent supported our hypothesis and might provide a practical intervention to improve reproductive outcomes for BPES patients with *FOXL2* mutations. However, research on granulosa cell coculture is still in its infancy. Further exploration of coculturing the immature oocytes with granulosa cells from normal donors in larger cohorts is warranted.

In conclusion, our study is the first to summarize the reproductive characteristics of women of reproductive age with BPES and demonstrates that these patients with *FOXL2* mutations have decreased ovarian reserve and fertility, as well as poor ART outcomes. The genotype-reproductive phenotype correlations were highly heterogeneous and cannot be generalized. Genetic counseling for fertility planning and preimplantation or prenatal genetic diagnosis to reduce offspring inheritance is recommended.

## Data Availability Statement

The original contributions presented in the study are included in the article/supplementary material. Further inquiries can be directed to the corresponding author.

## Ethics Statement

The studies involving human participants were reviewed and approved by Institutional Review Board of Center for Reproductive Medicine, Shandong University. The patients/participants provided their written informed consent to participate in this study.

## Author Contributions

XJ and YQ contributed to the study concept and design. TM, WZ, RZ, JL, and YG contributed to acquisition of data. TM, WZ, and XJ contributed to analysis and interpretation of data, and manuscript writing. All authors contributed to reviewing and approval of the final version of this work.

## Funding

This work was supported by the National Key Research & Developmental Program of China (2018YFC1003803), the National Natural Science Foundation of China (82171651, 82125014, 81971352), and the Young Scholars Program of Shandong University.

## Conflict of Interest

The authors declare that the research was conducted in the absence of any commercial or financial relationships that could be construed as a potential conflict of interest.

## Publisher’s Note

All claims expressed in this article are solely those of the authors and do not necessarily represent those of their affiliated organizations, or those of the publisher, the editors and the reviewers. Any product that may be evaluated in this article, or claim that may be made by its manufacturer, is not guaranteed or endorsed by the publisher.
